# Double Laser for Depth Measurement of Thin Films of Ice

**DOI:** 10.3390/s151025123

**Published:** 2015-09-29

**Authors:** Manuel Domingo Beltrán, Ramón Luna Molina, Miguel Ángel Satorre Aznar, Carmina Santonja Moltó, Carlos Millán Verdú

**Affiliations:** Centro de Tecnologías Físicas, Universitat Politècnica de València, 46022 Valencia, Spain; E-Mails: ralunam@fis.upv.es (R.L.M.); msatorre@fis.upv.es (M.A.S.A.); mcsanton@fis.upv.es (C.S.M.); cmillan@fis.up.es (C.M.V.)

**Keywords:** thin films, thickness, refractive index

## Abstract

The use of thin films is extensive in both science and industry. We have created an experimental system that allows us to measure the thicknesses of thin films (with typical thicknesses of around 1 µm) in real time without the need for any prior knowledge or parameters. Using the proposed system, we can also measure the refractive index of the thin film material exactly under the same experimental conditions. We have also obtained interesting results with regard to structural changes in the solid substance with changing temperature and have observed the corresponding behavior of mixtures of substances.

## 1. Introduction

A thin film is a layer of material that ranges in thickness from fractions of a nanometer (monolayer) to several micrometers. The use of thin films is widespread, with applications in many areas: in optics, oxides of Zr and Hf are used on glass substrates to reduce reflection losses and to increase the transmission of optical systems [1]; in food packaging, thin film coatings of Al are applied to polymeric substrates as protective layers to prevent oxygen exposure [2]; in solar cells, thin films are used in the fabrication of ZnO/n-Si junctions [3]; in astrophysical ices, films of simple molecules (CO_2_, CO, CH_4_, N_2_, NH_3_) with thicknesses of around 1 µm are deposited to enable characterization of these materials under conditions similar to those in astrophysical environments [4]; in spacecraft applications, protective coatings of Al_2_O_3_ and SiO_2_ are used to prevent low Earth orbiters from being damaged by the harsh environmental conditions [5]; and SiO_x_ coatings have been investigated for oxidation barrier applications to protect polymers from photo-oxidation [6].

Several techniques have been developed for growth of these films, and the most commonly used methods can be classed into several groups. These groups include: physical methods, such as evaporation and molecular beam epitaxy (MBE) [7]; chemical-physical methods, such as sputtering, plasma processes, and thermal formation processes [8]; gas-phase chemical methods, such as vapor phase chemical deposition, vapor phase epitaxy, and ion implantation; liquid phase chemical methods, such as electrodeposition and liquid phase epitaxy; and mechanical techniques [9].

For all the applications mentioned above, it is necessary to quantify the thickness of the thin film accreted. Film thickness is defined as the perpendicular distance from any point on a surface to the other side of the film. There are several different ways to measure thickness [10], including measurement with or without physical contact based on the measurement of optical characteristics when the film is complete, or even during film growth. The selection of an appropriate method depends on both the coating material (dielectric or conductive, opaque or transparent) and the order of magnitude of the thickness to be measured. Some of the most commonly used methods for thin film thickness measurement are described below.

### 1.1. Stylus Profilometer

**Figure 1:**
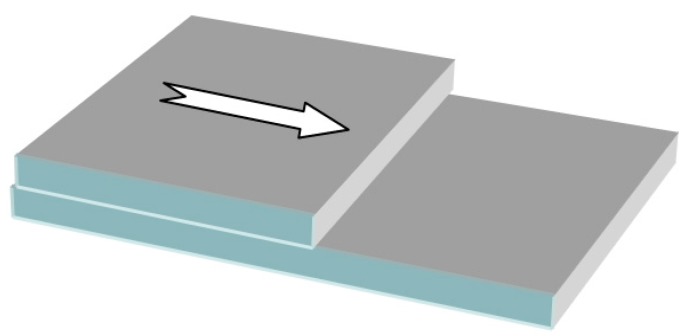
Application of stylus profiler to thin film thickness measurement.

The stylus profilometer is a mechanical instrument [11] that requires the presence of a step (Figure 1) or a groove between the substrate surface and the film surface. This discontinuity can be produced by masking portions of the substrate during deposition or by removing parts of the film after deposition. For this reason, this method is considered to be destructive. The stylus profilometer consists of a diamond-tip pin probe connected to a magnetic pickup coil. The vertical displacement of the probe along the horizontal path corresponds to the measured thickness. The measurement range starts from 20 Å and has no upper limit, and the accuracy of the method is <3%.

### 1.2. Ellipsometry

**Figure 2:**
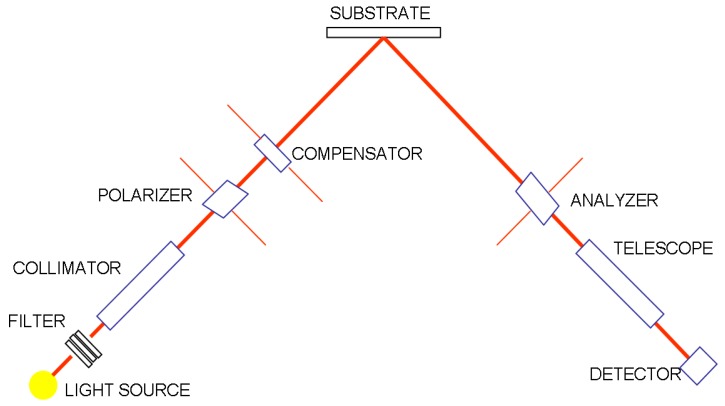
Diagram of thickness measurement by ellipsometry.

In ellipsometry [12], an incident circularly-polarized monochromatic beam is reflected by the film (Figure 2). The change in the polarization of the reflected beam provides information about the film’s thickness, refractive index and extinction coefficient via the expression $\rho =e^{i\Delta }\text{tan}\psi$, where ρ is the ratio of the Fresnel reflection coefficients for p (parallel) and s (perpendicular) polarized light, and Δ and ψ denote the relative changes in phase and amplitude, respectively, of the components p and s of the light beam after reflection. This measurement is performed after film growth is complete. The measurement range extends from a few Å up to a few µm, with an uncertainty of 1 Å.

### 1.3. Interferometry

**Figure 3:**
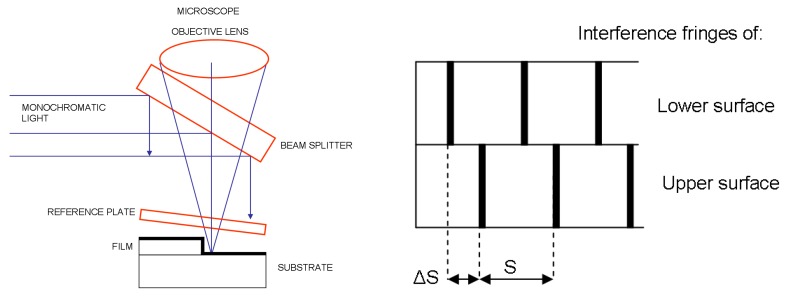
Diagram of monochromatic light interference-based thickness measurement process for opaque films.

Interferometry [13] uses the phase difference between two beams of a monochromatic light source to measure the thickness of thin films (Figure 3). This method, of which there are several different versions, can be considered nondestructive because does not alter the film structure.

For opaque thin films, a step is normally formed and the part of the exposed substrate and the surface of the film are covered with a highly reflective material. Using a reference plate, an air wedge is formed over each surface of the step. Two interference patterns are obtained, and the thickness is given by the expression $d=\frac{\Delta S\lambda }{S}$. The measurement range extends from 10 to 20000 Å, with an uncertainty of 2 Å.

**Figure 4:**
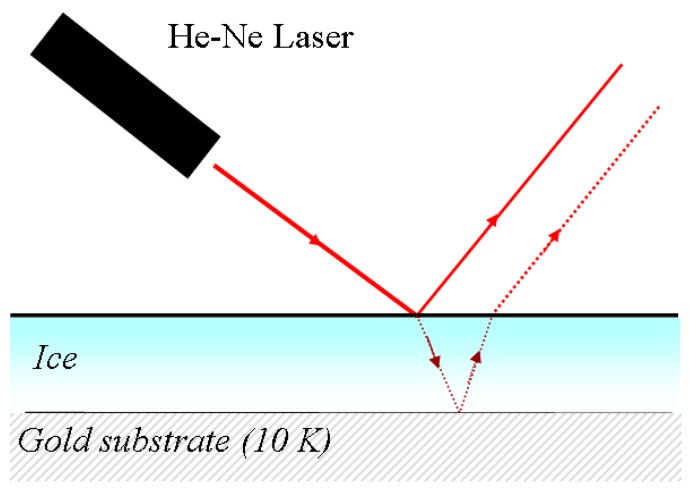
Diagram of single laser interference-based thickness measurement process for transparent films.

For transparent thin films, the interference originates from the reflection from the surface of the film and the reflection from the substrate after light transmission through the film (Figure 4). The interference of these two beams can then be used to determine the film thickness. The measurement range of the method extends from 800 Å to 10 µm, with precision of around 0.03%. This method is explained in more detail in the following sections.

### 1.4. Mass Measurement

An alternative way to calculate the thickness of a thin film is by measurement of its mass. If the deposited mass m, the area A and the density ρ are known, then the film thickness is $d=\frac{m}{A\rho }$. Normally, a quartz crystal microbalance (QCMB) is used to obtain the mass, using the Sauerbrey equation ∆F = S·∆m that shows the relationship between the variation of the mass ∆m and the variation of frequency ∆F of the oscillator that constitutes the QCMB, through the constant S. The result also depends on the accuracy of the film density, and the error is around 3%.

A specific version of the interferometric thickness measurement technique has been developed and implemented in our laboratory. Our method is based on the use of two lasers set at different angles of incidence to the growing film, which enables the thickness and the corresponding refractive index of the film to be obtained simultaneously. Otherwise, two different film deposition experiments would be required to obtain both parameters, but it would be impossible to ensure that both films had exactly the same growth conditions. In addition, because the interference can be monitored during film accretion, it is possible to control the film thickness in real time during growth using this method.

This paper describes the experimental procedure used to perform thickness measurements for thin films of ice. In the next section, the experimental setup is shown, and the procedure and fundamentals of the process are explained in the following section. In section 4, the results are reported, and finally we present our conclusions.

## 2. Experimental Section

**Figure 5:**
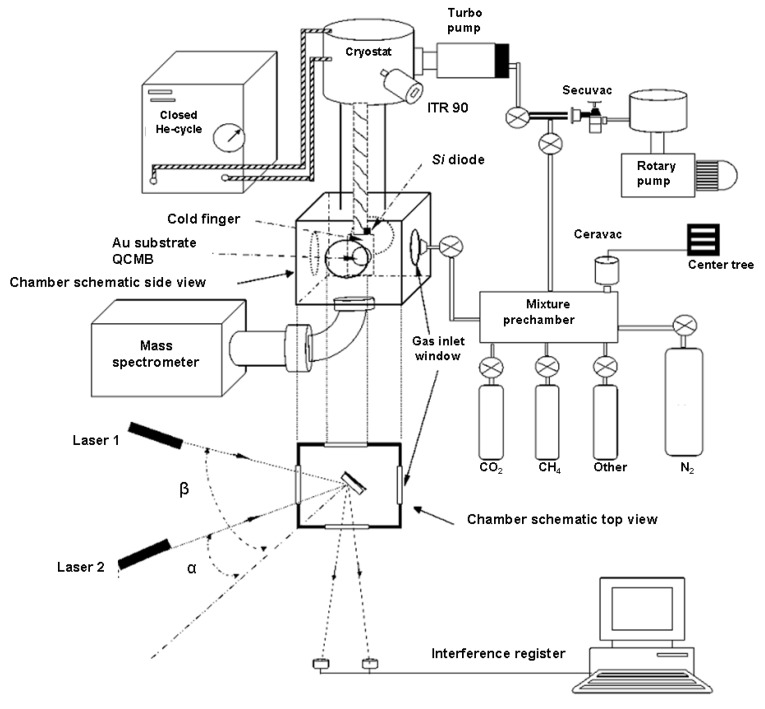
Setup of the experimental apparatus.

A schematic of our hardware setup is shown in Figure 5. The basic procedure for a typical experiment using double laser interferometry is as follows: the substance that will form the film for which the thickness is to be measured is introduced in the gas phase in a chamber under high vacuum (HV) conditions (10^−7^ mbar). Part of this gas solidifies when it contacts the cold substrate.

In our experimental apparatus, the main components used to measure the depth of the thin ice films are two He-Ne lasers (1205-2, JDS Uniphase Corporation, Milpitas, CA, USA), which operate at a wavelength of 632.8 nm. The laser signals are collected by two photodiodes (BPW21, Centronic, Croydon, NH, USA), which are connected to a data acquisition system (National Instruments, USB-6009, National Instruments Spain, Madrid, Spain) that is controlled using software developed in-house based on LabView that registers the signals of both diodes and of some other instruments simultaneously. The lasers are positioned at two different angles of incidence, which are selected to be as close as possible to the optimum values that were deduced and will be discussed later in this work; these values depend on the physical conditions of the equipment.

To ensure good purity during film accretion, film growth is performed under HV and low temperature conditions on a sample holder (70 mm high) placed within a suitable chamber (chamber volume: 2000 cm^3^). A high vacuum and low temperature system is thus implemented as part of our apparatus.

The vacuum in the chamber is obtained using two turbomolecular pumps (Leybold Turbovac 50 and TMH 071 Pfeiffer, Asslar, Germany), which are both backed up by corresponding rotary pumps (Edwards EV8 and Edwards E1M18, respectively, Edwards Iberica Vacuum, Barcelona, Spain). Additionally, a cryostat is used to obtain the desired vacuum, and is also used to cool the system; it is a closed-cycle He cryostat (RDK 10-320, Leybold Vakuum, GmbH, Cologne, Germany) operating in a Gifford-McMahon cycle, and it is placed in contact with the sample holder. This device acts in two stages, where the first stage reaches a temperature of 40 K, and the second stage is able to reach 13 K. The second stage of the cryostat (240 mm high) and the sample holder are in contact and are referred to collectively in this paper as the cold finger. The first stage of the cryostat is also used as a cryopump for the system, and helps the system to obtain a base pressure of less than 10^−7^ mbar when molecules are adsorbed by (or adhered to) the cold walls of the first stage.

The pressure in the chamber is measured using an ITR IoniVac Transmitter (5% in accuracy, Leybold Vakuum, GmbH, Cologne, Germany) and the pressures of each process gas are also monitored in the chamber using a quadrupole mass spectrometer (QMS, AccuQuad RGA 100, Kurt J. Lesker Company, Pittsburgh, PA, USA) with resolution of ~0.5 amu and ionization energy of 70 eV.

Because the film growth temperature is a relevant parameter, the temperature of the sample is controlled using the ITC 503S Intelligent Temperature Controller (Oxford Instruments, Abingdon, UK). The instrument uses the feedback from a silicon diode sensor (Scientific Instruments, Palm Beach County, FL, USA.) located just beside the quartz element, which allows the temperature to be varied between 12.0 and 300.0 K (±0.5 K) via a resistive heater. This device allows us to grow a film at a specific temperature or even to increase the temperature at a constant rate.

The velocity of the molecules when they enter the main chamber also affects the growing film. A prechamber has therefore been implemented to control the rate at which molecules enter the main chamber. In this prechamber, the pressure and the aperture of the needle valve (D50968, Leybold Vakuum, GmbH, Cologne, Germany) that is located between the two chambers combine to regulate the flow of molecules. The pressure is measured using a Ceravac CTR 90 (Leybold Vakuum, GmbH, Cologne, Germany), which has an accuracy of 0.2% and is based on a ceramic sensor that is not influenced by the gas type (range 104, FS 100 torr). The prechamber becomes even more relevant when it is necessary to grow the required film from a mixture of several gas species. The gas mixtures are formed in the prechamber, based on their partial pressures.

When the gas or gas mixture has been prepared in the prechamber, the needle valve is opened and the gas then fills the chamber and adheres to the sample holder, and also adheres wherever the temperature in the chamber is below the sublimation temperature. This implies deposition on the cold substrate coming from all parts of the chamber, which is known as background deposition.

The chemicals that are typically used in our research are around 99.997% pure (Air Products and Praxair). To preserve the gas purity, the entire prechamber is evacuated using a turbo-drag pumping station (ultimate pressure <10^−6^ mbar).

## 3. Procedure

Laser interferometers have been widely used to measure the thickness of thin films [14]. First we explain in detail the procedure used to perform thickness measurements using a single laser, and we will then show how the procedure changes when using two lasers. The latter case has the advantage of providing us with two parameters: the refractive index of the material and the measured thickness. However, when the measurement is performed with only a single laser, the refractive index must be known to enable calculation of the thickness. In any case, the lasers are arranged in such a way that the electric field is perpendicular to the plane of incidence; the Brewster angle therefore cannot be reached and the reflection intensity will be greater than if the electric field were parallel to the plane of incidence.

**Figure 6:**
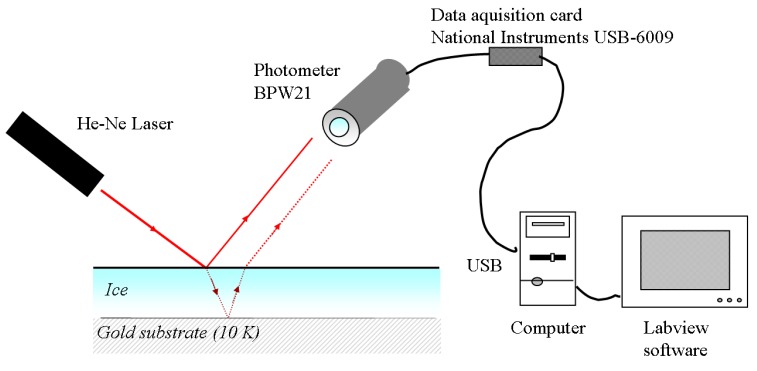
Scheme for thickness measurement using a single laser.

The measurement scheme based on a single laser is shown in Figure 6.

**Figure 7:**
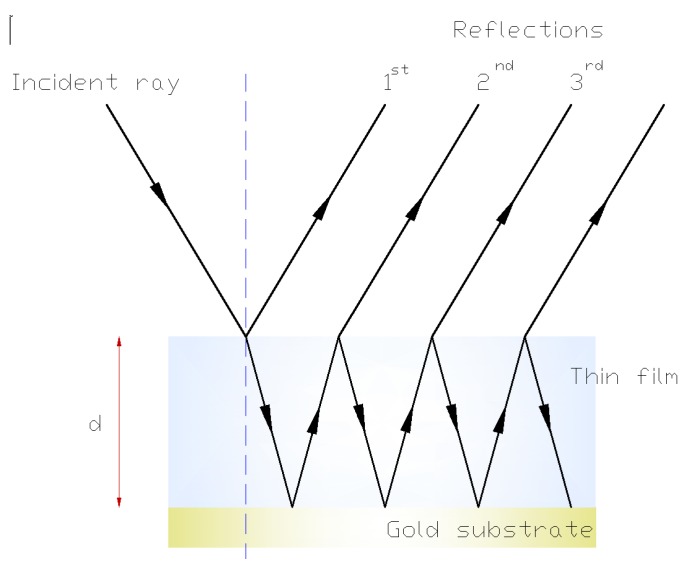
Multiple reflections in a thin film.

When the gas is introduced into the chamber, it becomes an ice layer that forms a transparent plane-parallel plate of thickness d and refractive index n. During film growth, a laser beam (λ = 632.8 nm, regarded as a plane wave in the direction indicated by the incident ray) is incident on the ice, and the beam undergoes both reflection and refraction at the first interface. The refracted wave is also reflected by the bottom surface, and then undergoes further reflections and refractions at the two surfaces, as shown in Figure 7.

Because multiple reflections are produced by the single incident wave, the superposition of these reflections in the photodetector gives an intensity that varies with the deposited film thickness, because the different phases produce alternating constructive and destructive interference.

**Figure 8:**
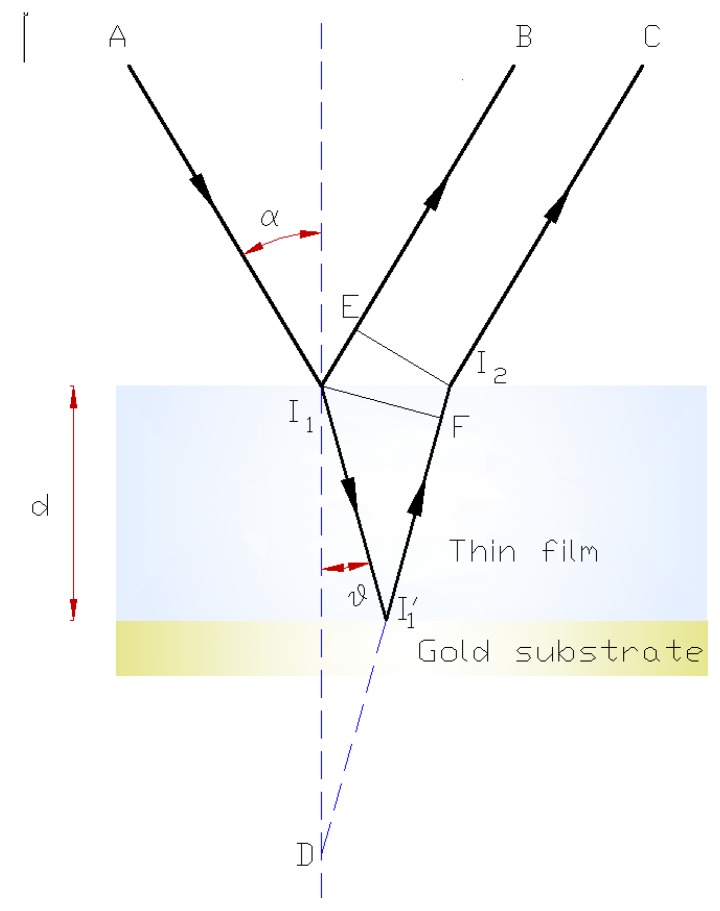
Interference ray diagram in a thin film.

In practice, only the first two reflections are taken into account, and to calculate the phase difference between them, it is sufficient to calculate the optical path difference [15] (Figure 8), because the reflections in both cases occur in the higher refractive index medium and the incident electric field is perpendicular to the plane of incidence.

The waves shown in Figure 8 are represented by the rays AI_1_B and AI_1_I’_1_I_2_C. No path difference is produced by the common wavefront I_2_E, and the path difference from point I_1_ to point I_2_E is: I_1_I’_1_I_2_ − I_1_E.

The corresponding optical path difference is Δ: Δ = n(I_1_I’_1_ + I’_1_I_2_) − n_o_I_1_E, where n is the refractive index of the film and n_o_ = 1 (for a vacuum or low-pressure gases). Because I_1_E = nI_2_F, we find that: Δ = n(I_1_I’_1_ + I’_1_F) = nDF = nI_1_Dcosθ = 2ndcosθ, where θ is the angle of refraction. The corresponding phase difference (δ) will therefore be:

(1) $$\delta =\frac{2\text{π}}{\lambda_{0}}\Delta =\frac{4\text{π}}{\lambda_{0}}\text{ndcosθ}$$

Constructive interference will occur when δ is an integer multiple of 2π:

(2) $$2\text{mπ}=\frac{4\text{π}}{\lambda_{0}}\text{ndcosθ},\text{ m}=1,\;2,\;\ldots ,\;i$$

The thickness d can therefore be isolated as

(3) $$d=\frac{{\text{mλ}}_{0}}{2\text{ncosθ}}$$

Between two consecutive maxima, where m = 1, the thickness is:

(4) $$d_{\alpha }=\frac{\lambda_{0}}{2\text{ncosθ}}$$

**Figure 9:**
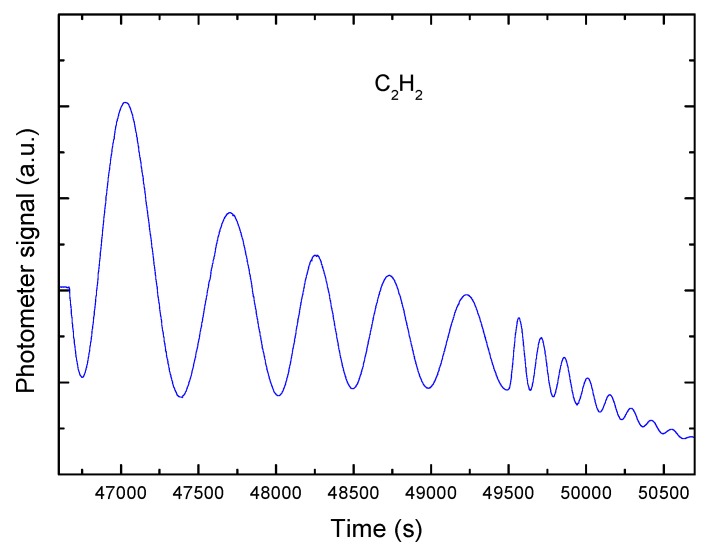
Graph of interference curves produced over time when using a single laser during gas-phase deposition.

In Figure 9, the interference pattern (intensity of the reflected signal *versus* time) obtained during a typical C_2_H_2_ deposition experiment is plotted. We can see the consecutive maxima and minima that were produced as the thickness increased. We can also observe how the signal intensity decreases with increasing deposition thickness.

This decreasing intensity profile is mainly produced by the following two factors:

(a) As the film is deposited, the surface roughness increases, which leads to increased scattering of the reflected light;

(b) As the film thickness increases, the surface becomes a lens-shaped surface rather than a flat surface, and this shape also affects the scattering of the reflected light.

Remarkably, the signal damping is not caused by absorption; if it was, the intensity would oscillate around a constant value of intensity with time. However, it can be seen that this is not the case, because the mean value decreases with time. It is also possible to distinguish two different zones within the interference pattern. In the left part of the pattern (composed of the first five maxima), the oscillations are more clearly separated in time, while in the right part (composed of the last eight maxima), the maxima are much less clearly separated in time. This occurs because two different pressures are used in the prechamber (where a higher pressure is required for the second part of the deposition), leading to different deposition velocities.

**Figure 10:**
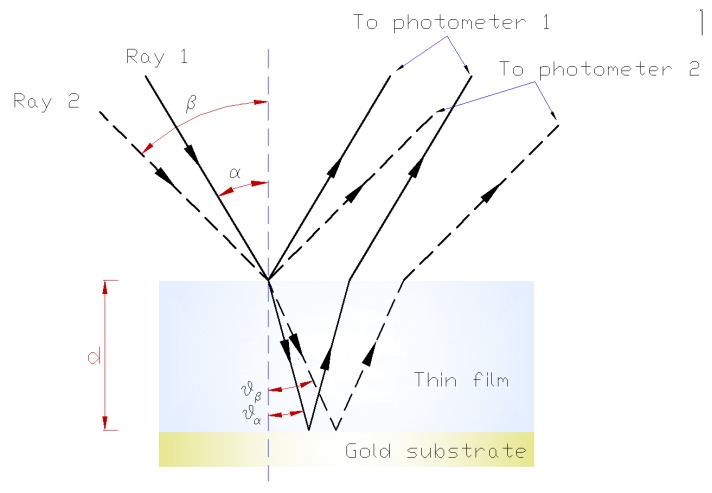
Ray diagram when using two laser beams.

From the Equation (4) that was given earlier to derive the thickness of a film when using only one laser, we see that the refractive index of the material must be known, and it is not always known a priori, particularly when mixtures of molecules are used during film accretion. However, if we use two lasers with different angles of incidence α and β (as shown in Figure 10), we obtain two equations and two unknowns (n and d).

When we take the Snell equation $n_{0}\cdot \text{sin}\alpha =n\cdot \text{sin}\theta_{\alpha }$ into account, and assuming that n_o_ = 1, we obtain

(5) $${\text{sinθ}}_{\alpha }=\frac{\text{sinα}}{\text{n}}$$

Using ${\text{sin}}^{2}{\text{θ}}_{\alpha }+{\text{cos}}^{2}{\text{θ}}_{\alpha }=1$ leads to:

(6) $${\text{cosθ}}_{\alpha }=\sqrt{\text{1}-\frac{{\text{sin}}^{2}\text{α}}{{\text{n}}^{2}}}$$

Therefore, the film thickness that is deposited during the time period that elapsed between two maxima is:

(7) $${\text{d}}_{\text{α}}={\frac{\text{λ}}{2\text{ncosθ}}}_{\text{α}}=\frac{\text{λ}}{2\text{n}\sqrt{1-\frac{{\text{sin}}^{2}\text{α}}{{\text{n}}^{2}}}}$$

This procedure allows us to change the angle of refraction in expression (4) as a function of the angle of incidence. To calculate the refractive index n, we perform a simultaneous assembly with the other laser using a different angle of incidence β, as shown in Figure 10, and we obtain

(8) $${\text{d}}_{\text{β}}=\frac{\text{λ}}{{2\text{ncosθ}}_{\text{β}}}=\frac{\text{λ}}{2\text{n}\sqrt{\text{1}-\frac{{\text{sin}}^{2}\text{β}}{{\text{n}}^{2}}}}$$

The quotients of both expressions give us:

(9) $$\left(\frac{{\text{d}}_{\text{α}}}{{\text{d}}_{\text{β}}}\right)=\frac{\text{λ}}{{2\text{ncosθ}}_{\text{α}}}\frac{{2\text{ncosθ}}_{\text{β}}}{\text{λ}}=\frac{\sqrt{\text{1}-\frac{{\text{sin}}^{2}\text{β}}{{\text{n}}^{2}}}}{\sqrt{\text{1}-\frac{{\text{sin}}^{2}\text{α}}{{\text{n}}^{2}}}}$$

**Figure 11:**
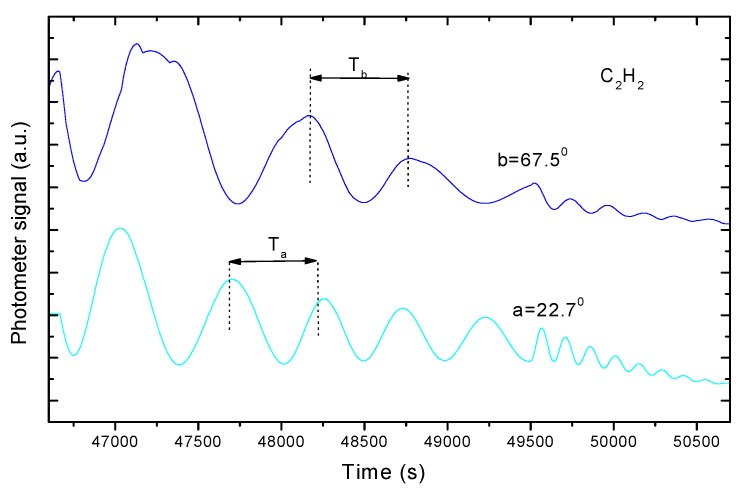
Graphs of interference curves produced over time with two lasers during gas-phase deposition.

For each laser beam, we can then write an equation that relates the change in thickness d between two maxima of the interference curve, the time T that elapsed between these maxima (measured as indicated in Figure 11) and the deposition rate v:

(10) $$\begin{array}{l} d_{\alpha }=v\cdot T_{\alpha }\\ d_{\beta }=v\cdot T_{\beta } \end{array}$$

Because v is the same in both expressions (which is a key aspect of our method), the resulting γ after division of the two expressions above can be calculated from the experimental data (Figure 8) using the following.

(11) $$\frac{d_{\alpha }}{d_{\beta }}=\frac{T_{\alpha }}{T_{\beta }}=\gamma ;\;\frac{d_{\alpha }}{d_{\beta }}=\frac{T_{\alpha }}{T_{\beta }}=\gamma =\frac{\sqrt{1-\frac{{\text{sin}}^{2}\beta }{n^{2}}}}{\sqrt{\text{1}-\frac{{\text{sin}}^{2}\alpha }{n^{2}}}}$$

In this way, the value of n can already be obtained in terms of the angles of incidence α and β:

(12) $$n^{2}=\frac{{\text{sin}}^{2}\beta -\gamma^{2}{\text{sin}}^{2}\alpha }{1-\gamma^{2}}$$

A priori, any values of α and β are suitable. However, an algorithm that can be used to select optimum values for these angles to minimize the uncertainty of the refractive index values that were calculated using the previous expressions will be given in the next section. Because a laser beam at a smaller angle of incidence provides a shorter period, this angle must be called α to obtain a quotient where γ < 1.

**Figure 12:**
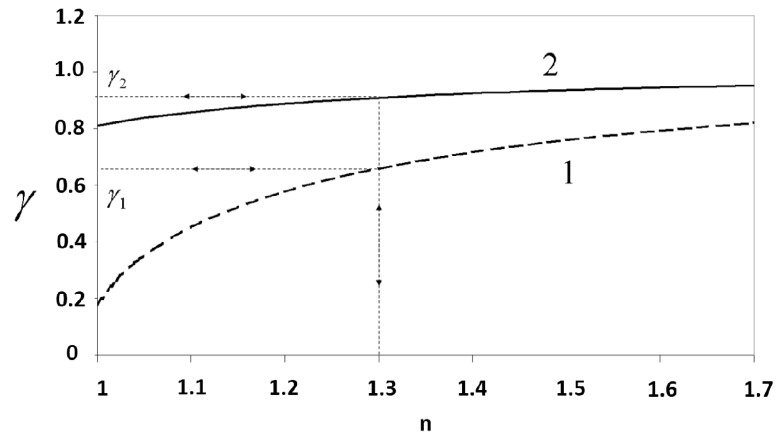
γ as a function of *n* for two different angle of incidence pairs: Curve 1: 10° and 80°; Curve 2: 33° and 47°.

Despite the fact that the growth rate is kept as constant as possible, experimentally-induced errors can alter the period of each laser, and the index value obtained may consequently be affected. To study the effects of these errors, Figure 12 plots the last equation for two different incident angle pairs.

The refractive index values of the ices in which we are interested may reach values close to 1.3. In the case of Curve 1, near the value of n = 1.3, the slope varies more sharply than that in Curve 2. For this reason, small variations in γ induced by unavoidable experimental errors could affect the results obtained with Curve 2 more strongly and thus yield unacceptable refractive index values.

**Figure 13:**
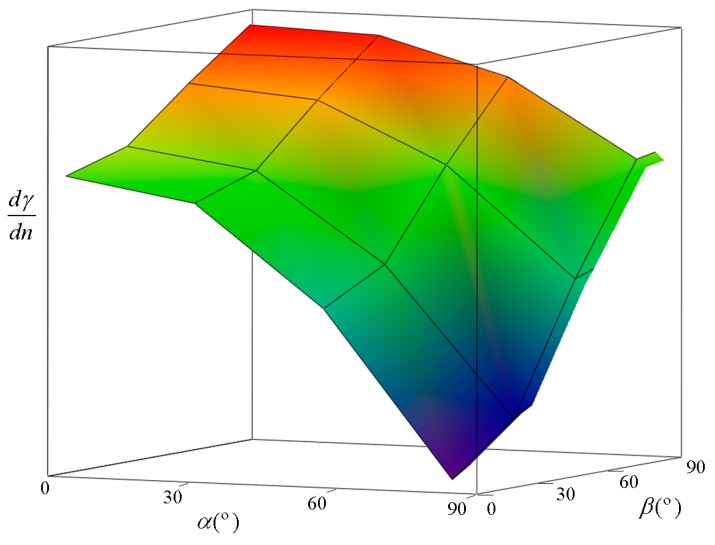
Derivative of γ *versus* refractive index at different angles of incidence. We can see that the highest value is reached when α is close to 0° and β is close to 90°.

Therefore, to reduce the uncertainty in determination of n, the previous condition is used to choose the incident angles such that the plot of γ as a function of *n* must have the greatest possible slope around the value of 1.3. To do this, we plot (in Figure 13) the derivative of Equation (11), in which γ and n are related, for n = 1.3 *versus* the available values of the incident angles α and β.

In Figure 13, we can see that the derivative (slope) reaches a maximum value when α and β are well separated, e.g., they are close to 0° and 90°, respectively. Therefore, the angles must be chosen by achieving this condition. In our case, the vacuum chamber and the other devices let us set the angles of incidence at 12.8° and 77.3°.

These angles are measured by basic trigonometry using distances of more than 5 m.

## 4. Results and Discussion

To validate our experimental setup, Table 1 compares our results with other results from the literature that were produced under similar conditions, and our results show good agreement with the previous results.

**Figure 14:**
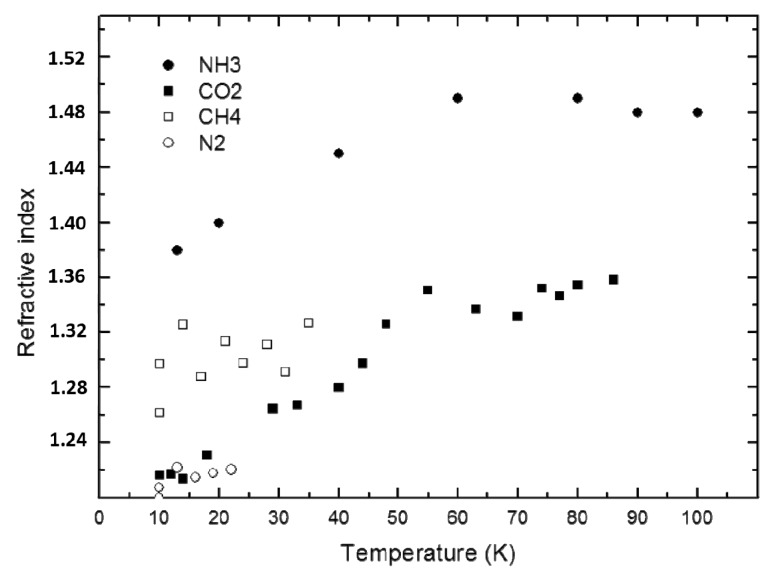
Refractive index values of CH_4_, N_2_, CO_2_ and NH_3_ for different gas-phase deposition temperatures.

Using this technique, our research group has performed several experiments in recent years to determine, among other quantities, the refractive index values of materials under astrophysical conditions. We have thus obtained some interesting experimental results. For some substances (e.g., CH_4_, N_2_), the refractive index value remains constant for different ice formation temperatures, while for other ices (CO_2_, NH_3_) the value varies [4,16], as shown in Figure 14. These results indicate possible structural differences with temperature in the types of ice where the refractive index changes [17,18].

**Table 1:** Comparison of our results with previous results from the literature, for λ=632.8 nm.

Authors	Molecule	Temperature (K)	Index of refraction
Shulze and Abe (1980) [17]	CO_2_	18	1.23
Satorre *et al.* (2008) (Us) [4]	CO_2_	18	1.23
Roux *et al*. (1980) [19]	N_2_	20	1.22
Satorre *et al.* (2008) (Us) [4]	N_2_	20	1.21
Roux *et al.* (1980) [19]	CH_4_	20	1.33
Satorre *et al.* (2008) (Us) [4]	CH_4_	20	1.30
Romanescu *et al.* 2010 [20]	NH_3_	80	1.49
Satorre *et al.* (2013) (Us) [16]	NH_3_	80	1.49

**Figure 15:**
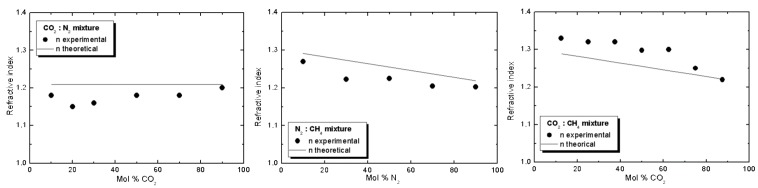
Refractive index values of binary mixtures for different gas-phase deposition temperatures.

We have also found that the experimentally measured refractive index for mixtures of molecules does not have a value that maintains the ratio of its components [21], and the refractive index value can be higher than expected (Figure 15). This is caused by interactions between the different types of molecules (adding new vibration modes to the responses of the matter to electromagnetic waves) and between the pure molecules.

## 5. Conclusions/Outlook

We have developed a suitable system that allows us to perform experiments on thin films at desired low temperatures. The basic magnitude used to characterize these films is their thickness. Our hardware allows us to measure this magnitude with a precision of 2.5% in real time. To enable us to control the process or to determine the substance that forms the thin film precisely, we simultaneously measure the refractive index of the substance. This is a very important aspect when using this procedure, because we do not need any prior data and we can check for the purity of the substance and for the absence of contamination.

**Figure 16:**
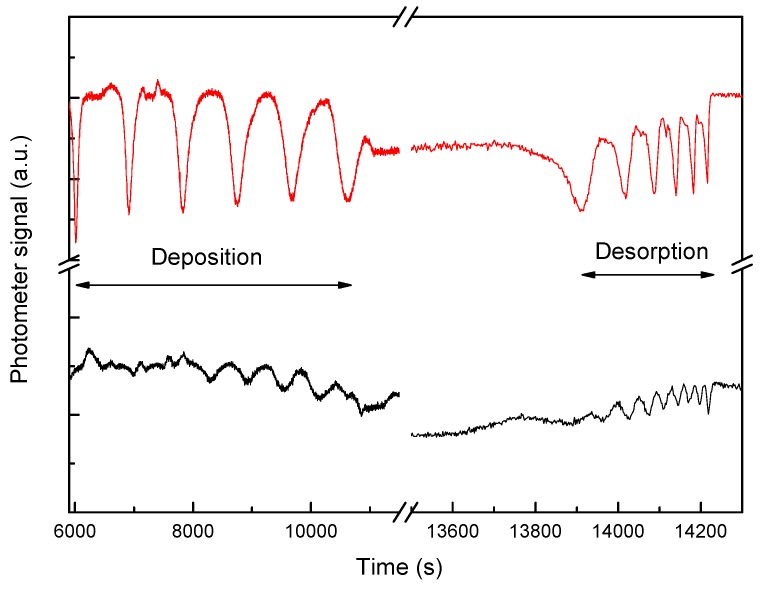
Complete process of thin film deposition and desorption when monitored using two lasers.

When the thin film is formed, a desorption process will begin if we increase the temperature. Our system is so sensitive that it is possible to register both the start and the end of this process (Figure 16). This physical phenomenon is related to the material structure and its porosity, and important material parameters can be obtained, such as the enthalpy of sublimation [22].

Other physical magnitudes that can be obtained using this experimental set up, when complemented with additional apparatus, include the density [4] and the polarizability [23] of each film.
